# From the Cochrane Library: Systemic Interventions for Steven-Johnson Syndrome (SJS), Toxic Epidermal Necrolysis (TEN), and SJS/TEN Overlap Syndrome

**DOI:** 10.2196/46580

**Published:** 2024-01-30

**Authors:** Gaurav Nitin Pathak, Thu Minh Truong, Amit Singal, Viktoria Taranto, Babar K Rao, Audrey A Jacobsen

**Affiliations:** 1 Department of Dermatology Rutgers Robert Wood Johnson Medical School Somerset, NJ United States; 2 Department of Dermatology Rutgers New Jersey Medical School Newark, NJ United States; 3 Department of Dermatology New York Institute of Technology Glenhead, NY United States; 4 Department of Dermatology Rao Dermatology Atlantic Highlands, NJ United States; 5 Department of Dermatology Hennepin Healthcare Minneapolis, MN United States; 6 Department of Dermatology University of Minnesota Minneapolis, MN United States; 7 Department of Dermatology Veterans Affairs Medical Center Minneapolis, MN United States

**Keywords:** Steven-Johnson syndrome, toxic epidermal necrolysis, necrolysis, fatal, life-threatening, treatment, dermatology, skin, dermatological, SJS, TEN, corticosteroids, intravenous immunoglobulin, IVIG, etanercept, prednisolone, systematic, corticosteroid, corticoid, steroid, steroids

## Introduction

Steven-Johnson syndrome (SJS), toxic epidermal necrolysis (TEN), and SJS/TEN overlap syndrome are a spectrum of potentially life-threatening, rare, and severe cutaneous adverse reactions that are triggered by medication use typically within weeks of medication initiation. The pathogenesis of SJS/TEN is theorized to be a T lymphocyte–mediated immune response to an antigen of the offending medication causing epidermal necrosis [1]. There is limited evidence to support the use of therapies, such as glucocorticoids, intravenous immunoglobulins (IVIGs), cyclosporine, and etanercept, for the treatment of SJS and TEN [1]. We aim to summarize the key findings of a Cochrane review on the effects of systemic therapies for SJS/TEN.

## Methods

To evaluate systemic therapies for SJS/TEN, a systematic review of randomized controlled trials (RCTs) and prospective observational comparative studies (up to March 2021) of patients of all ages with SJS/TEN was conducted [1]. The primary end points were disease-specific mortality (DSM) and adverse events leading to the discontinuation of systemic treatment therapy. Secondary end points included time to complete re-epithelialization, intensive care unit length of stay, total hospital length of stay, illness sequelae, and adverse events.

## Results

In total, 9 studies with a total of 308 patients from across 7 countries were included in the analysis, of which 3 were RCTs and 6 were prospective observational studies; 2 studies were included in a meta-analysis. The risk of bias for the three RCTs was respectively rated as high, moderate, and low; all the prospective comparative studies were rated as having a high risk of bias. The interventions that were assessed included systemic corticosteroids, tumor necrosis factor-α inhibitors, and others (Table 1).

The overall level of certainty for the parameters of interest was low, so most findings were “uncertain.” It was uncertain if corticosteroids had a higher risk of DSM versus no corticosteroids (relative risk [RR] 2.55, 95% CI 0.72-9.03). It was also uncertain if there was a difference between IVIGs and no IVIGs in terms of DSM (RR 0.33, 95% CI 0.04-2.91), time to re-epithelialization (mean difference −2.93, 95% CI −4.4 to −1.46 d), or length of hospital stay (mean difference −2.00, 95% CI −5.81 to 1.81 d). Etanercept did not significantly reduce DSM compared to corticosteroids (RR 0.51, 95% CI 0.16-1.63; *P*=.72), and serious adverse events, such as sepsis and respiratory failure, occurred in treatment with both groups. It was also uncertain if there was any difference between the cyclosporine and IVIG groups in terms of the risk of DSM (RR 0.13, 95% CI 0.02-0.98). A summary of other comparator studies is included in Table 2.

**Table 1:** Key characteristics of included trials.

Study (author, year)	Study design	Sample size, n	Intervention	Outcome measured
Azfar et al [2], 2010	Prospective observational study	40	Corticosteroids (dose unknown) vs supportive care	Disease-specific mortality
González-Herrada et al [3], 2017	Prospective controlled study	22	Cyclosporine (PO^a^ 3 mg/kg/d or IV^b^ 1 mg/kg/d until re-epithelialization, then taper off 10 mg/d every 48 h) vs IVIG^c^ (0.75 g/kg/d for 4 d; lower dose for renal insufficiency), systemic corticosteroids (37.5- to 100-mg prednisone equivalents for 4 d), or supportive care	All-cause mortality, expected death rate based on SCORTEN^d^, time to stabilization of BSA^e^ involvement, time to re-epithelialization start, and time to complete re-epithelialization
Han et al [4], 2017	Prospective comparator study	28	Plasmapheresis (1-time dose of 1000 mL of Ringer-Locke and 2-3 L of plasma at 1 L/h) vs IVIG or corticosteroids (unknown dose)	Hospital length of stay
Jagadeesan et al [5], 2013	Prospective comparator study	36	IVIG (0.2- to 0.5-g/kg cumulative dose over 3 d) and IV dexamethasone (0.1-0.3 mg/kg/d; tapered within 1-2 wk) vs IV dexamethasone (0.1-0.3 mg/kg/d; rapidly tapered within 1-3 wk)	Disease-specific mortality, AEs^f^ leading to discontinuation, other AEs, mean days to full skin healing, mean length of hospital stay, and illness sequelae
Kakourou et al [6], 1997	Prospective comparative study	16	Corticosteroids (methylprednisolone bolus 4 mg/kg/d for 2 d after fever subsided) vs supportive care only	Mortality
Paquet et al [7], 2014	Open-label randomized controlled trial	10	IV NAC^g^ in 5% glucose over 20-h period (150 mg/kg in 250 mL over first h; then 150 mg/kg in 500 mL for 4 h; and, lastly, 150 mg/kg in 1000 mL over 15 h) and IV infliximab (5 mg/kg over 2 h) vs NAC-only regimen (same as former)	Disease-specific mortality
Saraogi et al [8], 2016	Prospective observational study	43	IV corticosteroids, IVIG, and combination of corticosteroids and IVIG vs supportive care	Arrest of disease progression, time to re-epithelialization, and mortality
Wang et al [9], 2018	Open-label randomized controlled clinical trial	91	Subcutaneous etanercept 25 mg (50 mg if >65 kg) twice weekly until skin lesions healed (n=48) vs IV prednisolone 1-1.5 mg/kg/d until skin lesions healed (n=43)	Disease-specific mortality and other AEs
Wolkenstein et al [10], 1998	Double-blind randomized controlled trial	22	Thalidomide 200 mg BID^h^ PO × 5 d vs placebo at same dosing regimen	Disease-specific mortality

^a^PO: per os.

^b^IV: intravenous.

^c^IVIG: intravenous immunoglobulin.

^d^SCORTEN: Score for Toxic Epidermal Necrolysis.

^e^BSA: body surface area.

^f^AE: adverse event.

^g^NAC: N‐acetylcysteine.

^h^BID: twice per day.

**Table 2:** Summary of key study findings.

Comparison	Number of patients (number of studies)	Anticipated absolute effects (95% CI)	Relative effect (95% CI)	Certainty of evidence (GRADE^a^)
Corticosteroids vs supportive care	56 (2 OS^b^) [2,6]	DSM^c^: 91 per 1000 (supportive care) vs 232 per 1000 (corticosteroid); TTCR^d^: NR^e^; ICU-LOS^f^: NR; TH-LOS^g^: NR; AE/DC^h^: NR	DSM: 2.55 (0.72 to 9.03); TTCR: NR; ICU-LOS: NR; TH-LOS: NR; AE/DC: NR	Very low
IVIG^i^ and supportive care vs supportive care	36 (1 OS) [5]	DSM: 55 (6 to 386) per 1000 (IVIG) vs 167 per 1000 (supportive care); TTCR: mean 10.93 d, mean difference 2.93 d lower (4.4 d lower to 1.46 d lower); ICU-LOS: NR; TH-LOS: mean 15.33 d, mean difference 2.00 d lower (5.81 d lower to 1.81 d higher); AE/DC: NR	DSM: 0.33 (0.04 to 2.91); TTCR: NR; ICU-LOS: NR; TH-LOS: NR; AE/DC: NR	Very low
Etanercept vs supportive care	No studies fit criteria	N/A^j^	N/A	N/A
Cyclosporine vs supportive care	No studies fit criteria	N/A	N/A	N/A
IVIG vs corticosteroids	No studies fit criteria	N/A	N/A	N/A
Etanercept vs corticosteroids	91 (1 RCT^k^) [9]	DSM: 163 per 1000 (corticosteroids) vs 83 (26 to 265) per 1000 (etanercept); TTCR: NR; ICU-LOS: NR; TH-LOS: NR; AE/DC: NR	DSM: 0.51 (0.16 to 1.63); TTCR: NR; ICU-LOS: NR; TH-LOS: NR; AE/DC: NR	Low
Cyclosporine vs corticosteroids	No studies fit criteria	N/A	N/A	N/A
Etanercept vs IVIG	No studies fit criteria	N/A	N/A	N/A
Cyclosporine vs other treatments (IVIG: n=4; corticosteroids: n=1; no specified treatment: n=1)	22 (1 OS) [3]	DSM: 500 per 1000 (other treatments) vs 65 (10 to 468) per 1000 (cyclosporine); TTCR: NR; ICU-LOS: NR; TH-LOS: NR; AE/DC: NR	DSM: 0.13 (0.02 to 0.98); TTCR: NR; ICU-LOS: NR; TH-LOS: NR; AE/DC: NR	Very low
Etanercept vs cyclosporine	No studies fit criteria	N/A	N/A	N/A
N-acetylcysteine and infliximab vs infliximab alone	10 (1 OS) [7]	NR	DSM: 2.00 (0.26 to 15.62)	NR
Thalidomide vs placebo	22 (1 RCT) [10]	NR	DSM: 2.78 (1.04 to 7.40)	NR
Plasmapheresis vs other treatments	28 (1 OS) [4]	NR	TH-LOS: mean difference −7.37 (−16.09 to 1.35) d	NR

^a^GRADE: Grading of Recommendations, Assessment, Development, and Evaluation.

^b^OS: observational study.

^c^DSM: disease-specific mortality of Steven-Johnson syndrome and toxic epidermal necrolysis.

^d^TTCR: time to complete re-epithelialization.

^e^NR: not reported.

^f^ICU-LOS: intensive care unit length of stay.

^g^TH-LOS: total hospital length of stay.

^h^AE/DC: adverse effects leading to discontinuation of Steven-Johnson syndrome/toxic epidermal necrolysis therapy.

^i^IVIG: intravenous immunoglobulin.

^j^N/A: not applicable.

^k^RCT: randomized controlled trial.

## Discussion

The authors of the original review concluded that “etanercept (25 mg [50 mg if weight > 65 kg]) twice weekly ‘until skin lesions healed’) may reduce DSM compared to corticosteroids (intravenous prednisolone 1 to 1.5 mg/kg/day ‘until skin lesions healed’) (RR 0.51, 95% CI 0.16 to 1.63; 1 study; 91 participants; low‐certainty evidence); however, the CIs were consistent with possible benefit and possible harm” [1]. Overall, data from the included studies were limited, with few direct clinical comparator studies for the different therapeutic agents assessed. Future multicenter large-scale studies are needed to better outline SJS/TEN medication therapy and evaluate agents of choice in disease management.

## Abbreviations

DSM: disease-specific mortality
IVIG: intravenous immunoglobulin
RCT: randomized controlled trial
RR: relative risk
SJS: Steven-Johnson syndrome
TEN: toxic epidermal necrolysis
